# Three-Dimensional Reconstruction Modeling of the Spatial Displacement, Extent and Rotational Orientation of Undisplaced Femoral Neck Fractures

**DOI:** 10.1097/MD.0000000000001393

**Published:** 2015-10-02

**Authors:** Xin Fu, Gui-Jun Xu, Zhi-Jun Li, Chang-Ling Du, Zhe Han, Tao Zhang, Xinlong Ma

**Affiliations:** From the Department of Orthopedics, Tianjin Hospital, Tianjin, P.R. China (XF, G-JX, ZH); Department of Orthopedics, General Hospital of Tianjin Medical University, Tianjin, P.R. China (Z-JL, TZ); and Department of Orthopedics, Binzhou Medical University Hospital, Shandong, P.R. China (C-LD).

## Abstract

The purpose of this study was to employ a new three-dimensional (3D) reconstruction and modeling method to measure displacement of undisplaced femoral neck fractures (Garden stages I and II). We also aimed to evaluate the effectiveness of the Garden classification for determining the displacement of undisplaced femoral neck fractures.

A total of 120 consecutive patients with undisplaced femoral neck fractures were enrolled between 2012 and 2014, including 60 within the Garden I group and 60 within the Garden II group. The displacements of the femoral head center (d1) and the lowest point of the fovea capitis femoris (d2) and rotational displacement of the femoral head (α) in the 3D model were measured with 3D computed tomography reconstruction and modeling. Five observers, trauma surgeons, were asked to found the centers of the femoral heads and the deepest points of the foveae. The intraobserver and inter-observer agreements were calculated using Fleiss’ kappa.

The inter-observer and intra-observer kappa values were 0.937 and 0.985, respectively. Current method has good reliability. We discovered that many participants in our study had been misclassified by an anterior–posterior radiograph as having an “incomplete” fracture. In incomplete fracture of Garden stage I group, the average displacements d1 and d2 were 3.69 ± 1.77 mm and 14.51 ± 1.91 mm, respectively. The mean α was 4.91° ± 2.49°. For impacted fracture of Garden stage I, significant spatial displacement in the impacted fractures was observed (d1: 6.22 ± 3.36 mm; d2: 10.30 ± 5.73 mm; and α: 17.83° ± 10.72°). Similarly, significant spatial displacement was observed among the Garden stage II group (d1: 7.16 ± 4.58 mm; d2: 12.95 ± 8.25 mm; and α: 18.77° ± 9.10°). There was no significant difference in α, d1, and d2 between impacted fracture and Garden stage II groups (*P* > 0.05). However, significant differences were found between incomplete fracture and Garden stage II groups (*P* < 0.05).

Our findings suggest that 3D reconstruction and modeling may be a better tool for assessing femoral neck fractures than the Garden classification. Undisplaced femoral neck fractures showed variable degrees of displacement and were not undisplaced, stable fractures. Garden classification for undisplaced femoral neck fractures has certain limitations.

## INTRODUCTION

The worldwide aging of the population has led to a marked increase in the prevalence of hip fractures, as the most important risk factor for these fractures is advancing age.^1,2^ These injuries are of critical concern to the medical community, as they impose heavy economic burden on healthcare systems; current global predictions suggest that the cost of femoral neck fractures will increase from 1.7 million in 1990 to 6.3 million by the year 2050.^3,4^

Despite its increasing global prevalence, there is still much uncertainty in the medical community regarding the most efficient tools for the assessment and treatment of femoral neck fractures. A more comprehensive understanding of the spatial displacement (displaced or undisplaced) of femoral neck fractures would enable the medical community to treat these injuries more efficiently.^5^ At present, there is no gold standard for the treatment of undisplaced femoral neck fractures; much controversy still lies in whether these fractures should be treated through surgical operational or without surgical intervention.^6^

The Garden classification is often used to classify femoral neck fractures. Through the use of an anterior–posterior (AP) radiograph, femoral neck fractures are categorized into 4 classes based on the displacement of the fracture: stage I-incomplete fracture, abducted or impacted; stage II-complete fracture without displacement; stage III-complete fracture with partial displacement; and stage IV-complete fracture with full displacement.^7–9^ Although it is the most widely used for treatment decisions, the Garden classification has several disadvantages: it is poorly reproducible; it offers low inter-rater reliability; and it is not considered to have any prognostic value.^9,10^ The development of a reasonable and effective method for measuring the spatial displacement of the femoral head is now of critical concern.^11^

Evidence is building that a computed tomography (CT) may allow for more accurate diagnosis of the displacement of femoral neck fractures. Though a CT may offer some advantages to the use of an AP radiograph, it is not yet clear if a CT can practically measure the spatial displacement in femoral neck fractures.^12^ Given the lack of a sensitive and specific indicator of femoral neck fractures, we have proposed a new method to describe the position space transfer of femoral head by using CT images to construct a 3D reconstruction and model of the fracture.^13^ The purpose of present study is to measure the spatial displacement in undisplaced femoral neck fractures by using 3D reconstruction and digital technology. A specific aim of this study is to re-evaluate Garden's classification, in order to inform future practice in the assessment and treatment of femoral neck fractures.

## METHODS

Participants were recruited from the Department of Orthopedics and Traumatology at Tianjin Hospital from January 2012 to June 2014, following admission to the hospital with an undisplaced femoral neck fracture (Garden stage I or II). Our available sample included 462 males (44.4%) and 578 females (55.6%) and the mean age was 75 years of age (range: 26–88). Participants were excluded from the study if they had: displaced femoral neck fracture; ipsilateral femoral shaft fracture or bilateral femoral neck fractures; pathological lesions in proximal femur; congenital malformation; or a history of fracture in the studied hip. The present study received approval from the ethics committee of Tianjin Hospital; informed written consent was obtained from all participants.

### Fracture Assessment

AP radiographs were reviewed independently by 2 orthopedic surgeons (ZH and XF), as well as a radiologist (Z-JL). The distinction between the undisplaced and displaced femoral neck fractures was made using the Garden's system with an AP radiograph. In order to minimize inter-rater bias, we distributed the Garden classification criteria to all the authors. In the event that the readers failed to come to an agreement, the assessment of a traumatologist (X-LM) was accepted as the final decision.

In addition to AP radiographs, further axial images of the pelvis and proximal femur were obtained using a multislice CT scanner (Siemens Healthcare, Forchheim, Germany), with a slice thickness and pitch set to 0.75 mm. The CT was set to 120 kVp and 100 mAs to produce an image matrix of 512 × 512 pixels and a field of view of 100 mm. All CT image were obtained in Digital Imaging and Communications in Medicine (DICOM) format for the purpose of reconstruction.

The Medical Image Processing and Visualization software Mimics 10.01 (Materialise, Leuven, Belgium) was used for image modeling and reconstruction. The 2-dimensional (2D) slices of both sides of proximal femur DICOM files were automatically combined and converted to 3D models. A mirror 3D model of the fractured side was superimposed onto the model of the contralateral normal femur.

**FIGURE 1:**
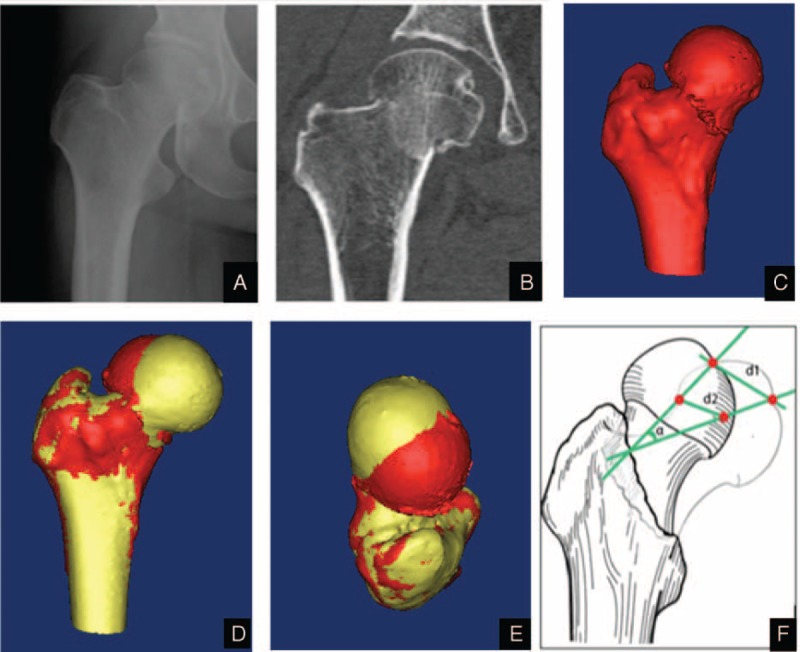
A Garden stage I right femoral neck fracture. (A) Antero-posterior radiograph. (B) Coronal scanning of computed tomography. (C) 3-dimensional model of femoral neck fracture. (D) Front view of bilateral femoral 3-dimensional model. (E) Top views of bilateral femoral 3-dimensional model. The red model represents femoral model with fracture, and the yellow model represents the healthy femoral model. (F) The figures describe the new measuring method for spatial displacements.

**FIGURE 2:**
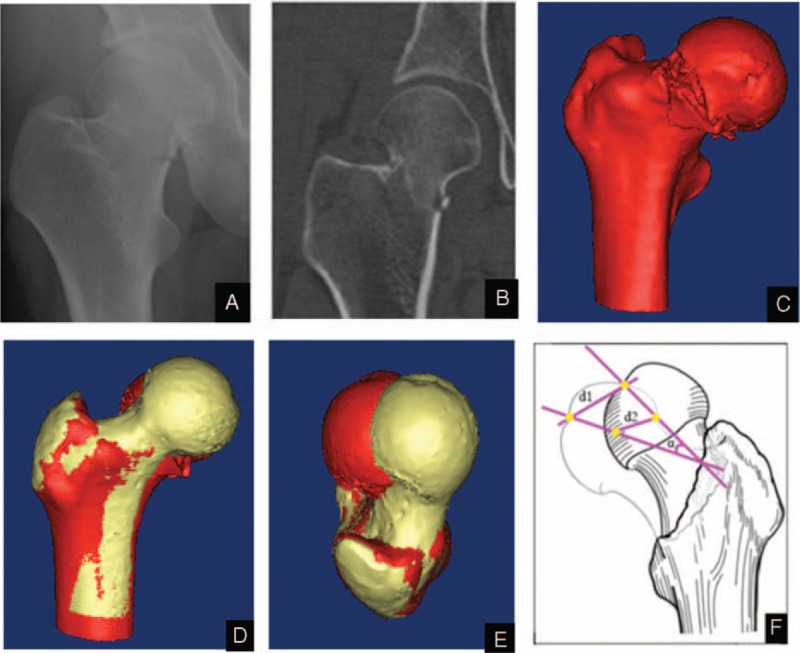
A Garden stage II right femoral neck fracture. (A) Antero-posterior radiograph. (B) Coronal scanning of computed tomography. (C) Three-dimensional model of femoral neck fracture. (D) Front view of bilateral femoral 3-dimensional model. (E) Top views of bilateral femoral 3-dimensional model. The red model represents femoral model with fracture, and the yellow model represents the healthy femoral model. (F) The figures describe the new measuring method for spatial displacements.

The displacement and rotation of the undisplaced femoral neck fractures were measured using 2 distinct and constant anatomical landmarks: the femoral head center; and the deepest point of the fovea of the femoral head. The center of the femoral head was defined using computer-aided design software after a close-fit sphere was built up at the edge of the femoral head.^14^ Two parameters were selected to describe the displacement between healthy and mirrored fractured femoral heads was described by 2 parameters: d1 (the distance between the centers of the femoral heads) and d2 (the deepest points of the foveae). A best-fit line was drawn from the center of the femoral head to the deepest point of the fovea. The angle (α) of the best-fit line from the mirrored fractured model to the healthy model was selected to represent the rotation of the femoral heads in undisplaced femoral neck fracture. Figures 1 and 2 show this method. The CT data were reviewed by 5 reviewers (XF, G-JX, Z-JL, C-LD, and ZH). Each reviewer was asked to found the centers of the femoral heads and the deepest points of the foveae. Two weeks after completion of the initial review process, they were asked to find the same anatomical landmark again.

### Statistical Methods

All statistical analyses were performed using SPSS 20.0 software (SPSS, Inc., Chicago, IL). Inter-observer and intra-observer agreements were determined by performing weighted kappa coefficient calculation. According to Landis and Koch, kappa coefficients <0 indicate no agreement; 0.0 to 0.2, slight agreement; 0.21 to 0.4, fair agreement; 0.41 to 0.6, moderate agreement; 0.61 to 0.8, substantial agreement; and 0.81 to 1.0, almost perfect agreement.^15^ Descriptive statistics were used to summarize sample demographics and were presented in terms of means and standard deviations. A Mann-Whitney *U* test, the nonparametric alternative to the independent-samples *t* test, assessed differences in the mean values for d1, d2, and α by Garden stage group (I vs II). We considered *P* values of 0.05 or less to indicate statistical significance.

## RESULTS

Of the available sample (n = 1040), 60 (5.8 %) participants within the Garden stage I group and 60 (5.8 %) participants within the Garden stage II group were chosen for further analysis. Within the Garden stage I group, 55% participants (33 of 60) had right femur fractures, 53% participants (35 of 60) were female and the mean age was 73 (range: 27–81 years of age). Similarly, within the Garden stage II group, 51.7% participants (31 of 60) had right femur fractures, 61.7% participants (37 of 60) were female and the mean age was 75 (range: 42–85 years of age). All patients underwent surgery (internal fixation or arthroplasty). The median follow-up period was 20.3 months. The inter-observer and intra-observer kappa values were 0.937 and 0.985, respectively. Current method has good reliability.

### Garden Stage I Group

**FIGURE 3:**
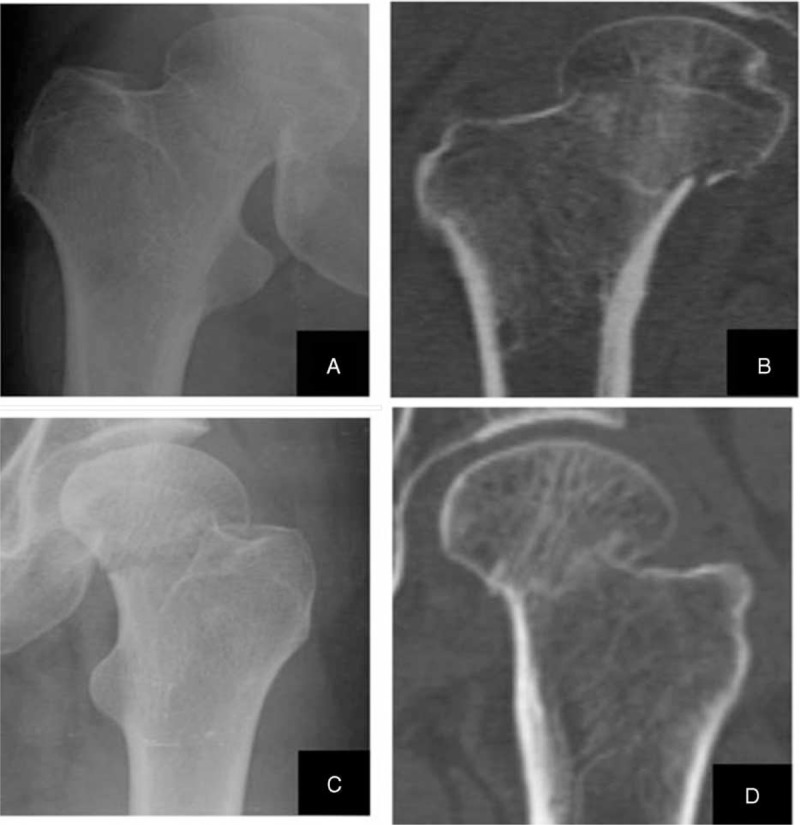
(A) Antero-posterior radiograph of a right hip showing an incomplete femoral neck fracture. (B) Coronal scanning of computed tomography showing a complete fracture. (C) Antero-posterior radiograph of a left hip showing a Garden stage I femoral neck fracture. (D) Coronal scanning of computed tomography shows an impacted femoral neck fracture.

Upon examining the reconstructed models in Garden stage I group, we discovered that 9 of the 15 incomplete fractures were classified as “complete” according to the AP radiographs. In incomplete fracture of Garden stage I group, the average displacements d1 and d2 were 3.69 ± 1.77 mm and 14.51 ± 1.91 mm, respectively. The mean α was 4.91° ± 2.49°. For impacted fractures classified by radiographs, significant spatial displacement was observed (Figure 3). Twelve patients underwent internal fixation and none of them had ONFH, other 3 patients underwent arthroplasty. In impacted fracture of Garden stage I group, the average displacements d1 and d2 were 6.22 ± 3.36 and 10.30 ± 5.73 mm, respectively. The displacement of the femoral head center exceeded 10 mm in more than half of the participants (33 of 60 participants). Rotational displacement of the fractures was not readily observed using the CT scans alone; however, 3D reconstructed models showed marked rotational displacement of the femoral head. The mean α was 17.83° ± 10.72° and a rotational displacement of 10° to 50° was observed among 80% of the participants. Thirty-eight patients underwent internal fixation and 3 of them had ONFH, other 7 patients underwent arthroplasty.

### Garden Stage II Group

All Garden stage II fractures were classified as complete but not displaced on the anteroposterior radiographs; however, 3D reconstruction revealed that all fractures had a spatial displacement (Figure 3). Compared to incomplete fracture of Garden stage I group, the spatial displacement parameters measured by AP radiographs were much higher. All of the femoral neck fractures showed rotational displacement. The mean d1 and d2 were 7.16 ± 4.58 and 12.95 ± 8.25 mm, respectively. The mean α was 18.77° ± 9.10°. An α > 20° was observed among 41.7% (25 of 60) of the participants. Forty-seven patients underwent internal fixation and 4 of them had ONFH, other 13 patients underwent arthroplasty.

### Comparison on Garden Stage Groups

**FIGURE 4:**
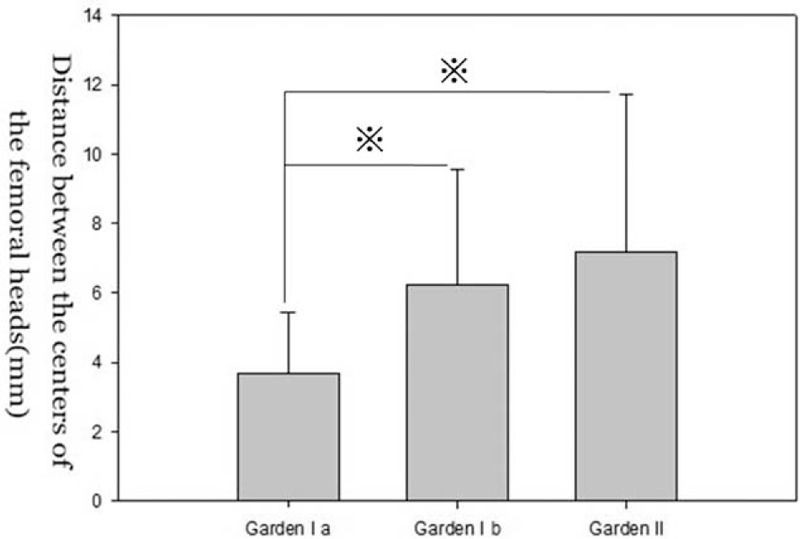
Graph showing displacement of the center of femoral head (d1) in patients with undisplaced femoral neck fractures. The values of d1 are smaller in incomplete fracture group (Garden Ia) than other 2 groups. There was no significant difference in the d1 between impacted fracture group (Garden Ib) and complete fracture group (Garden II).

**FIGURE 5:**
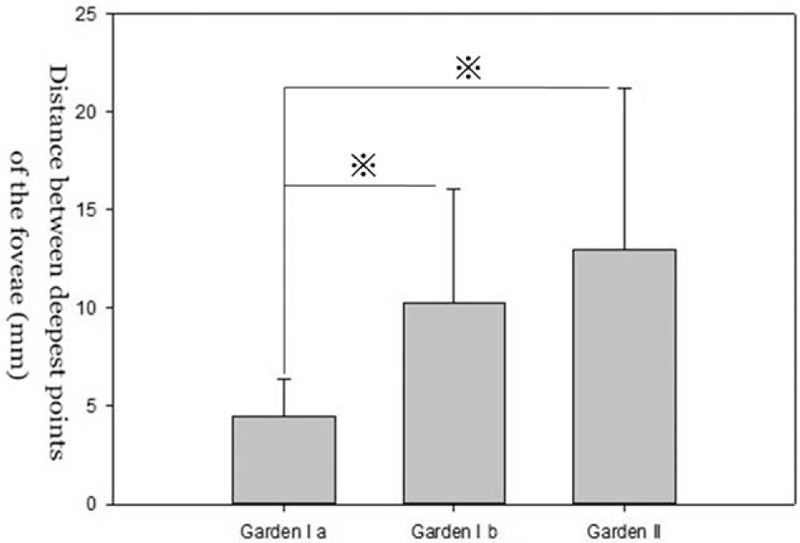
Graph showing displacement of the lowest point of femoral head fovea (d2) in patients with undisplaced femoral neck fractures. The values of d2 are smaller in incomplete fracture group (Garden Ia) than other 2 groups. There was no significant difference in the d2 between impacted fracture group (Garden Ib) and complete fracture group (Garden II).

**FIGURE 6:**
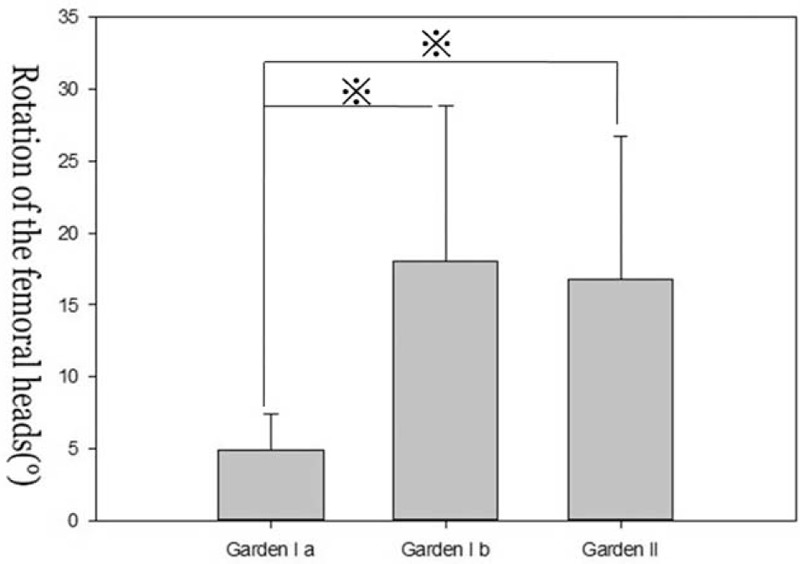
Graph showing rotational displacement of the femoral head (α) in patients with undisplaced femoral neck fractures. The values of α are smaller in incomplete fracture group (Garden Ia) than other 2 groups. There was no significant difference in the α between impacted fracture group (Garden Ib) and complete fracture group (Garden II).

We compared the means for α, d1, and d2 across the Garden stage groups using a Mann-Whitney *U* test. There was no significant difference in α, d1, and d2 between impacted fracture and Garden stage II groups (*P* > 0.05). However, significant differences were found between incomplete fracture and Garden stage II groups (Figures 4–6).

## DISCUSSION

The increased morbidity and mortality of undisplaced fractures associated with the femoral neck demands the immediate attention of the medical community.^16,17^ Fracture classification systems are considered as useful tools for making a decision on an adequate method of treatment and for disease prognosis evaluation.^7^ The Garden classification represents the most popular system currently used in clinical practice.^8^ Over its course of use in clinical settings, the reliability, reproducibility and ability to inform treatment procedures of the Garden classification has been brought to question.^9,10,18,19^

CT scanning has been preoperative routine examination for femoral neck fractures. In our studies, all CT data were obtained from preoperative routine examination. Results from our study demonstrated that the 3D reconstruction and modeling may be a better tool for assessing femoral neck fractures than an AP radiographs or 2D CTs. Specifically, we believe that the 3D modeling and reconstruction offer 3 distinct advantages to the use of an AP to evaluate, diagnose, and treat femoral neck fractures: increased precision in assessing spatial displacement; extent and rotational orientation of displacement; and time-efficiency.

### Increased Precision in Assessing Spatial Displacement

Perhaps the greatest disadvantage of the Garden classification is its inability to accurately assess the spatial displacement of the femoral head, a characteristic that is essential to the informing of treatment and reduction of clinical complications.^13^ Zlowodzki et al found that orthopedic surgeons were able to easily differentiate undisplaced and displaced fractures but were not as successful indistinguishing between the 4 classes of the Garden classification.^9^ It is pertinent to note that greater number of examiners had reported the total rate of reoperation, complications and mortality was significantly higher for displaced fractures than for undisplaced fractures.^6,11,16,20^ Thus, some have suggested that the system be collapsed into 2 categories: femoral neck fractures without displacement (Gardens I and II) and femoral neck fractures with displacement (Gardens III and IV).^6,21^

The displacement of femoral neck fractures is an important characteristic to consider when developing a treatment plan. Some advocate for nonoperative treatment of these injuries, noting that these fractures are often incomplete and stable.^22,23^ Complications (eg, osteonecrosis, secondary displacement, or pseudoarthrosis) are quite common following operative interventions; it is estimated that between 24% and 50% experience these complications.^24–27^ For instance, Gjertsen et al observed nonunion in 20% of patients and avascular necrosis in 3% at the 1-year follow-up for surgical intervention of undisplaced fractures.^15^ Furthermore, the reoperation rate for the undisplaced fractures is between 11% and 19%, due to fracture healing complications.^6,16,28^

Evidence is building that a CT scan better detect subtle comminution and detailed displacement than an AP radiograph.^29,30^ Katz et al claimed that a CT may be more useful for understand the invasion of distal radius fractures resulted in increased inter-rater reliability in the proposed management of these injuries and improved the sensitivity of measurement of articular surface gapping and comminution.^5^ Lasanianos et al reported that CT scan was more appropriate means to verify the hidden fractures and predicted the further complications in femoral neck fracture.^32^ An inherent limitation of the 2D CT is that sequential 2D images skip disruptions in the parallel radiographic planes; this often makes the interpretation of the 2D images confusing or inerrant. For this reason, the authentic displacement of the femoral head is in 3D reconstruction offers advantages to the 2D approach and should be further investigated.^33^

The use of 3D CT may offer improved intra-rater and inter-rater agreement, as well as improved sensitivity, specificity, and accuracy for the detection of spatial displacement.^34–36^ For instance, Gose et al found that 3D reconstructions was beneficial for the analysis of the femoral offset, the neck-shaft angle and the femoral anteversion of individuals with cerebral palsy,^34^ while Li et al found that 3D reconstructions were useful for measuring the antero-lateral coverage of femoral head.^33^ It is our belief that 3D CTs are the most accurate and direct method available for the assessment of spatial displacement in femoral head fractures.

### Extent and Rotational Orientation of Displacement

A major finding from our study in the discovery that 9 of 15 participants in the Garden stage I group had been misclassified by an AP radiograph as having an “incomplete” fracture. Others have noted the inerrant nature of AP radiographs in identifying the extent of the femoral neck fractures^13^ and is has been suggested that these fractures involve variable degrees of displacement.^37^ Ideally, a 3D reconstruction would be used to differentiate the extent of the displacement.^37^

As compared to an AP radiograph, 3D reconstruction and modeling are capable of measuring the rotational displacement of femoral neck fractures. In the present study, nearly half of the participants in the Garden stage I group showed a great rotational displacement of the femoral head in the 3D model; however, the AP radiograph was unable to detect the rotational orientation of the fracture. For the Garden stage II group, we detected prominent rotational displacement and spatial displacement of the femoral head in all 60 participants.

Aside from its use in the evaluation of femoral neck fractures, a solid understanding of the extent and rotational orientation of displacement is critical to the therapeutic strategy and prognosis of individuals with femoral neck fractures. Beyond the improvement in the assessment and detection of femoral neck fractures, these characteristics are crucial to the prediction of postoperative complications^11^ and reoperations.^16^ However, these previous studies are plagued with confounding bias due to the dependence on radiographs to determine the extent and rotational orientation, which offer unacceptable intra- and inter-rater reliability (Tables 1 and 2).

**TABLE 1:**
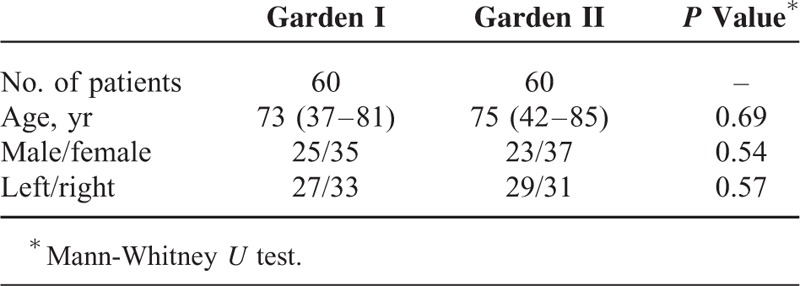
Baseline Characteristics

**TABLE 2:**
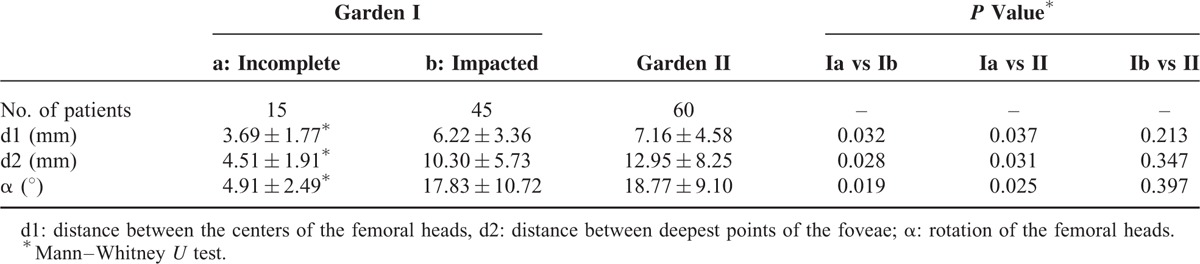
Data of Displacement and Rotation

We believe that Garden stages I and II fractures, particularly impacted fractures, classified by AP radiographs may be minimally displaced fractures, as opposed to stable or “undisplaced” fractures. Consequently, we suspect that this may lead to an increase in the number of fractures being classified within a higher Garden class (eg, stage III or IV). This is plausible, considering that the Garden classification was originally designed to focus on the trabeculae within the acetabulum and the femoral head, which do not sufficiently describe the without additional imaging perspectives. We believe that this warrants a correction to the Garden classification to include 2 new substages for undisplaced fractures: Garden Ia, Incomplete fracture; Garden Ib, Impacted fracture; Garden II, Complete fracture with minor displacement. This adjustment to the traditional Garden classification has been further supported in contemporary researchers.^9^

### Strengths and Limitations

The use of CT imaging introduced an increased risk of radiation exposure risk to the participants. Although radiation exposure of patients is a concern, we believe that controlled and limited doses of irradiation are acceptable to detect the accurate femoral displacement instead of requiring repeat scanning or radiation by 2D imaging. Howard et al indicated that only a small percentage of performed patient examinations triggered a notification or alert event from CT, the impact on workflow of adopting these features was negligible, following low dose exposure CT technology widely used, the radiation hazard can be reduced.^38^

Another limitation of our study exists in the relatively small sample size; we acknowledge that further studies with larger sample size are needed to confirm our current findings.

This study was designed to help develop and quick and reliable method for assessing displacement for physicians, nurses, and technicians. Although this objective is still novel, our study did include the measurement of Garden stage I or II of the femoral neck fractures. In further studies, we plan to investigate the association between spatial displacement and fracture stage. Additionally, our study did not directly evaluate whether the use of 3D CT scan better inform treatment plans or predict outcomes; further studies are needed to investigate explicitly determine these relationships.

## IMPLICATIONS

Despite methodological limitations, our study provides evidence for the appropriate use of 3D reconstruction and digital measurement technologies for assessment of undisplaced femoral neck fractures. This method provides more accuracy in evaluating the spatial displacement, extent and rotational orientation of the fracture than 2D examinations. Additionally, this study highlights a new quick and efficient method to accurately understand the true spatial displacement and rotation in undisplaced femoral neck fractures, which could guide orthopedists in making preoperative plans and offer reasonable treatment options to individuals with femoral neck fractures.
